# Psychosocial Factors and Quality of Life of Portuguese Adolescents With Chronic Conditions – Increased Risk for Victims of Bullying

**DOI:** 10.5334/cie.131

**Published:** 2024-10-15

**Authors:** Ana Cerqueira, Fábio Botelho Guedes, Tania Gaspar, Emmanuelle Godeau, Celeste Simões, Margarida Gaspar de Matos

**Affiliations:** 1 Institute of Environmental Health (ISAMB)/Aventura Social/Faculty of Medicine, University of Lisbon (FMUL), Lisbon, Portugal; 2 Faculty of Human Kinetics –University of Lisbon/FMH-UL, Lisbon, Portugal; 3 Catholic Research Center for Psychological, Family and Social Wellbeing (CRC-W), Faculty of Human Sciences, Portuguese Catholic University, Lisbon, Portugal; 4 Digital Human-Environment Interaction Labs (HEI-LAB), Lusófona University of Humanities and Technologies, Lisbon, Portugal; 5 French School of Public Health, EHESP, Rennes, France; 6 CERPOP –UMR 1295, unitémixte UMR INSERM –UniversitéToulouse III Paul Sabatier –Team SPHERE, Toulouse, France; 7 APPSYci/ISPA, Lisbon, Portugal

## Abstract

The experience of living with a chronic condition (CC) impacts adolescents’ psychological and social adjustment and overall functioning. Considering the increased risk of psychosocial challenges among adolescents with CC, this study aimed to enhance our understanding of the psychological and social factors that impact their quality of life. It also compared the psychological and social variables among (a) adolescents with and without CC and (b) adolescents with CC who are and who are not victims of bullying. The results demonstrated that adolescents with CC showed more psychosocial difficulties than their peers, as they more frequently reported involvement in situations of violence, and demonstrated more difficulties at a psychological and emotional level. Further, being victims of bullying increased the psychosocial vulnerabilities of these adolescents. A better quality of life was associated with the following psychosocial factors: not being a victim of bullying or cyberbullying, having less anxiety and fewer depressive symptoms, liking school, receiving more support from family and friends, having better relationships with peers, and having fewer physical and psychological symptoms. These findings are significant for helping schools develop tools and strategies to address violence and support students with CC, who are at a higher risk of being involved in such situations and require a targeted response.

## Characteristics of Adolescents With Chronic Diseases

Chronic or non-communicable diseases are characterized by the need for monitoring and management throughout the life cycle, as they are long-lasting health conditions with a generally slow progression (World Health Organization [WHO], 2018, 2020). The term “chronic disease” encompasses diverse health conditions with considerable variation (Bernell & Howard, 2016). For example, Stein et al. (1993) defined chronic conditions (CC) as health issues stemming from biological, psychological, or cognitive factors, persisting for at least one year and resulting in one or more of the following outcomes: developmental limitations, heightened reliance on third-party assistance, medication, or medical device dependence, and necessitating continuous medical care and treatment.

The experience of living with a CC tends to have psychological and social repercussions (Butler et al., 2018; Runions et al., 2020), affecting psychosocial adjustment and functioning (Gaspar et al., 2019; Malkowska-Szkutnik & Mazur, 2019; Mullins et al., 2017; Runions et al., 2020; Secinti et al., 2017; Sentenac et al., 2022; van der Sprenkel et al., 2022). In terms of psychological functioning, a diagnosis of CC may be associated with situations of stress, anxiety, and depression, among other problems (Revenson & Hoyt, 2016). Therefore, adolescents with CC have a higher risk of developing mental health problems (Butler et al., 2018; Runions et al., 2020).

Regarding social functioning, adolescents with CC exhibit an increased risk of involvement in bullying situations (Faith et al., 2015; Haegele & Zhu, 2023; Jackson et al., 2019; James et al., 2022; Pinquart, 2017; Pittet et al., 2010; Runions et al., 2021; Sentenac et al., 2011). Bullying is an aggressive and offensive behavior perpetuated for a continued period towards individuals who are at a disadvantage compared to the perpetrator of this behavior (Hellström et al., 2021). It is common among adolescents with subsequent consequences and repercussions on their physical health, psychosocial functioning, and academic performance (Arslan et al., 2021; Chudal et al., 2021; Eyuboglu et al., 2021; González-Cabrera et al., 2020; Jackson et al., 2019; Koyanagi et al., 2019; Li et al., 2022).

Adolescents with CC also tend to experience more difficulties in school than their peers (Cerqueira, Gaspar, et al., 2022; James et al., 2022; Kirkpatrick, 2020; Malkowska-Szkutnik & Mazur, 2019). Specifically, a CC influences various aspects of a student’s life, such as academic success, school attendance, relationships with teachers and peers, and participation in different social contexts (Kirkpatrick, 2020; Nap-van der Vlist et al., 2020, 2021; Runions et al., 2020; Schlebusch et al., 2020; Schlecht et al., 2023).

A study by Sentenac et al. (2022) that analyzed the school experience across different dimensions (including peer victimization) of adolescents from 19 European countries found that students with CC had higher levels of negative school experiences than their peers. This included school absenteeism resulting from the specificities of the existing health condition and lack of support appropriately adjusted to their needs (Malkowska-Szkutnik & Mazur, 2019; Pittet et al., 2010; Runions et al., 2020, 2021; Sentenac et al., 2013; Taylor et al., 2008).

Adolescents’ well-being is influenced by their family, school, and peer group, which can either support or threaten their health and well-being (Butler et al., 2022). Promoting school belonging and building positive relationships with peers and teachers can significantly impact adolescents’ overall well-being. That is, positive relationships create a sense of belonging and security which, in turn, contribute to a more fulfilling experience during the formative years (Buratta et al., 2023; Kirkpatrick, 2020).

With regard to bullying, in addition to the type of dynamics and the quality of relationships existing in the school environment, it is essential to consider the influence of the family and peer group environments on adolescents’ involvement in bullying (Ahmed et al., 2022; Biswas et al., 2022; Marini et al., 2023; Pouwels & Garandeau, 2021; Thornberg et al., 2022).

The level of participation of adolescents with CC in the different contexts of their lives (e.g., school, family, peer group) also affects their quality of life and well-being (Cerqueira, Gaspar et al., 2022; Cerqueira, Guedes, Marques-pinto et al., 2022). In fact, a higher risk of impairments in or barriers to social participation and the physical and psychological characteristics associated with their existing health condition are some of the factors that can contribute to a greater propensity of these students to be victims of bullying (Pinquart, 2017).

Evidence has also shown students with CC to be aggressors in bullying situations (Beckman et al., 2020; Haegele & Zhu, 2023; Pinquart, 2017; Rupp et al., 2019). Therefore, it is crucial to explore the risk factors associated with involvement in bullying (both as victims and aggressors) and quality of life among adolescents with CC to identify preventive and interventional strategies adjusted to their characteristics and needs (Godeau et al., 2022; Koyanagi et al., 2019).

Many studies have shown the impact that involvement in bullying has on the well-being and quality of life of adolescents (Ahmed et al., 2022; Arslan et al., 2021; Chudal et al., 2021; Eyuboglu et al., 2021; González-Cabrera et al., 2020; Jackson et al., 2019; Koyanagi et al., 2019; Li et al., 2022; Sentenac et al., 2022; Skarstein et al., 2020). However, much less is known about the impact of bullying specifically on adolescents with CC.

## Purpose of the Present Study

Given the increased risk of psychosocial challenges among adolescents with CC, the present study (a) compared psychological and social variables among adolescents with and without CC; (b) assessed differences in psychological and social variables between adolescents with CC who are and who are not victims of bullying; and (c) analyzed the psychological and social factors impacting their quality of life.

Based on the literature, we hypothesized that (a) adolescents with CC would present worse psychosocial outcomes than their peers without CC; (b) adolescents with CC who are victims of bullying would present worse psychosocial outcomes than adolescents with CC who are not bullied; and (c) better psychosocial functioning would be associated with a better quality of life. The psychological variables considered in the study consisted of self-harm, physical and psychological symptoms, anxiety, and depression. The social variables included bullying and cyberbullying, support from family and friends, liking school, and relationships with teachers and peers.

## Method

This work was based on the Health Behavior in School-aged Children (HBSC) 2018 study (Inchley et al., 2016; Matos & Equipa Aventura Social, 2018). The HBSC is a collaborative study by the World Health Organization (WHO) that follows a rigorous international protocol (Inchley et al., 2016; Roberts et al., 2009). Developed every four years the HBSC/WHO aims to analyze and understand adolescents’ health and risk behaviors in different contexts and how these behaviors affect their well-being.

The HBSC 2018 study in Portugal had the approval of the Ethics Committee of S. João do Porto Hospital and MIME (School Survey Monitoring). The study included 42 groups of schools, and parents or legal guardians gave informed consent regarding their children’s participation. Data from the adolescents were collected through an online self-report questionnaire, respecting the anonymity of all participants.

Further details about the data collection procedures for the HBSC 2018 study in Portugal are available in Matos and Equipa Aventura Social (2018).

### Participants

This study included 8,215 adolescents, 52.7% female, and 16.3% (*n* = 808) having a CC. The participants were from the 6th, 8th, 10th, and 12th year of schooling, aged between 10 and 22 years old, with an average age of 14.36 (*SD* = 2.28). The sample was proportionally distributed across five regions of mainland Portugal (North, Center, Lisbon and Tagus Valley, Alentejo, and Algarve).

### Variables and Measures

Table 1 presents the variables considered in this study.

**Table 1:** Variables and Measures Under Study.


VARIABLES	MEASURES

Chronic condition	1 – Yes; 2 – No

Being a bully	1 – No; 2 – Yes

Being a victim of bullying	1 – No; 2 – Yes

Being a cyberbully	1 – No; 2 – Yes

Being a victim of cyberbullying	1 – No; 2 – Yes

Liking school	1 – Dislike; 2 – Like

Self-harm	1 – No; 2 – Yes

Family support	Scale with four items rated on a seven-point Likert scale, with 1 being “very strongly disagree” and 7 being “very strongly agree.” Higher values reveal greater family support. *α* = .95.

Support from friends	Scale with four items rated on a seven-point Likert scale, with 1 being “very strongly disagree” and 7 being “very strongly agree.” Higher values reveal greater support from friends. *α* = .93

Relationship with teachers	Scale with three items rated on a five-point Likert scale, with 1 being “strongly agree” and 5 being “strongly disagree.” Higher values reveal a better relationship with teachers. *α* = .84.

Relationship with peers	Scale with three items, on a five-point Likert scale, with 1 strongly agree and 5 strongly disagree. Higher values reveal a better relationship with peers. *α* = .76.

Physical and psychological symptoms	Scale with nine items (back pain; neck pain, headache, dizziness; stomach pain; nervousness, irritation or bad mood, sadness and fear) rated on a five-point Likert scale (1 = “almost every day” and 5 = “rarely or never”). Minimum score of 9 and maximum of 45). Higher values reveal fewer symptoms. *α* = .82.

Anxiety	Scale with four items rated on a five-point Likert scale, with 1 being “never” and 5 being “always/almost always.” Higher values reveal higher levels of anxiety. *α* = .44.

Depressive symptoms	Scale with 10 items that assess depressive symptoms (e.g., feeling depressed, lack of hope, fear, loneliness, perception of unhappiness) rated on a five-point Likert scale, with 1 being “rarely or never” and 5 being “always or all the time. “Higher values reveal more depressive symptoms. *α* = .81.

Quality of life	Scale with ten items with scores from 0 to 5. Minimum scores of 5 and maximum scores of 50. Higher values reveal a better perception of quality of life. *α* = .83.


### Data Analysis

The data were analyzed using the Statistical Package for the Social Sciences (SPSS), version 27 for IOS. Descriptive statistics were performed for all variables under study (Tables 2 and 3). A chi-square test was used to analyze differences in distribution (a) by existing health condition (i.e., having/not having a CC); and (b) involvement in bullying situations as a victim (i.e., being/not being a victim) and the following psychosocial variables: involvement in bullying and cyberbullying situations (as victim and aggressor), liking school, and self-harm. Further, independent samples *t*-tests were used to analyze differences according to (a) existing health condition and (b) involvement in bullying situations as a victim and the remaining psychosocial variables under study: family support, support from friends, relationship with teachers, relationship with colleagues, physical and psychological symptoms, anxiety, depressive symptoms, and quality of life.

**Table 2:** Bivariate Analysis of Differences Between Students With and Without CC.


	*M ± SD* or % (*N*)			χ^*2/t*^	*p*

	TOTAL (*N* = 8,215)	CHRONIC CONDITION YES 16.3% (*n* = 808)	CHRONIC CONDITION NO 83.7%(*n* = 4,150)		

Being a bully^1^				0.263	0.608

No	90.4 (7041)	89.2 (721)	89.8 (3728)		

Yes	9.6 (752)	10.8 (87)	10.2 (422)		

Being a victim of bullying^1^				5.908	≤0.01

No	82.2 (6402)	80.0 (646)	**83.5** (3464)		

Yes	17.8 (1390)	**20.0** (162)	16.5 (686)		

Being a cyberbully^1^				0.156	0.693

No	94.7 (7380)	94.1 (760)	94.4 (3918)		

Yes	5.3 (411)	5.9 (48)	5.6 (232)		

Being a victim of cyberbullying^1^				4.780	≤0.05

No	91.9 (7160)	89.5 (723)	**91.8** (3811)		

Yes	8.1 (630)	**10.5** (85)	8.2 (339)		

Liking school^1^				1.116	0.291

Dislike	30.4 (2393)	33.3 (269)	35.2 (1462)		

Like	69.6 (5473)	66.7 (539)	64.8 (2688)		

Self-harm^1^				9.229	≤0.01

No	82.0 (4042)	78.2 (627)	**82.7** (3415)		

Yes	18.0 (890)	**21.8** (175)	17.3 (715)		

Family support^2^	23.87 ± 6.44	22.45 ± 6.85	23.32 ± 6.57	–3.437	≤0.001

Support from friends^2^	21.96 ± 6.75	21.99 ± 6.41	21.90 ± 6.66	0.366	0.715

Relationship with teachers^2^	11.28 ± 2.52	10.73 ± 2.43	10.91 ± 2.51	–1.892	0.059

Relationship with peers^2^	11.80 ± 2.41	11.73 ± 2.62	11.67 ± 2.46	–1.815	0.070

Physical and psychological symptoms^2^	36.74 ± 7.11	33.37 ± 7.95	36.29 ± 7.24	–10.321	≤0.001

Anxiety^2^	11.02 ± 2.67	11.50 ± 2.73	10.93 ± 2.64	5.316	≤0.001

Depressive symptoms^2^	18.05 ± 5.50	19.27 ± 5.95	17.81 ± 5.37	6.416	≤0.001

Quality of life^2^	36.43 ± 7.28	35.20 ± 7.13	36.68 ± 7.28	–5.295	≤0.001


^1^Chi-square; ^2^Independent sample *t*-test. **Adjusted residuals >1.96**.

**Table 3:** Bivariate Analysis of the Differences Between Being or Not Being a Victim of Bullying (Students With CC).


	*M* ± *SD* or % (*N*)		χ^*2/t*^	*p*

	BEING A VICTIM OF BULLYING YES 20.0% (*n* = 162)	BEING A VICTIM OF BULLYING NO 80.0% (*n* = 646)		

Being a bully^1^			56.675	≤0.001

No	72.8 (118)	**93.3** (603)		

Yes	**27.2** (44)	6.7 (43)		

Being a cyberbully^1^			28.558	≤0.001

No	85.2 (138)	**96.3** (622)		

Yes	**14.8** (24)	3.7 (24)		

Being a victim of cyberbullying^1^			51.091	≤0.001

No	74.1 (120)	**93.3** (603)		

Yes	**25.9** (42)	6.7 (43)		

Liking school^1^			4.258	≤0.05

Dislike	**40.1** (65)	31.6 (204)		

Like	59.9 (97)	**68.4** (442)		

Self-harm^1^			31.147	≤0.001

No	61.9 (99)	**82.2** (528)		

Yes	**38.1** (61)	17.8 (114)		

Family support^2^	19.32 ± 8.20	23.23 ± 6.24	6.672	≤0.001

Support from friends^2^	18.66 ± 7.36	22.83 ± 5.87	7.657	≤0.001

Relationship with teachers^2^	10.05 ± 2.77	10.90 ± 2.31	4.011	≤0.001

Relationship with peers^2^	10.06 ± 3.18	11.86 ± 2.32	8.120	≤0.001

Physical and psychological symptoms^2^	30.59 ± 8.29	34.07 ± 7.71	5.047	≤0.001

Anxiety^2^	12.51 ± 2.47	11.25 ± 2.73	–5.083	≤0.001

Depressive symptoms^2^	21.86 ± 6.30	18.63 ± 5.69	–5.803	≤0.001

Quality of life^2^	31.62 ± 7.48	36.10 ± 6.76	7.371	≤0.001


^1^Chi-square; ^2^Independent sample *t*-test. **Adjusted residuals >1.96**.

A multiple linear regression model was also developed to explore the impact of the psychosocial variables under study on the quality of life of adolescents with CC. This model was adjusted for age and gender.

A significance level of *p* < 0.05 was determined in all the analyses performed.

## Results

Table 2 contains the characteristics of the participants and the results of the bivariate analysis of the differences between students with and without CC with regard to the psychosocial variables under study. As illustrated, statistically significant differences were found related to being a victim of bullying and cyberbullying, self-harm, family support, physical and psychological symptoms, anxiety, depressive symptoms, and quality of life. That is, adolescents with CC reported more frequently being victims of both bullying and cyberbullying, engaging in self-harm behavior, and experiencing higher levels of anxiety and more depressive symptoms. In contrast, adolescents without CC reported more family support, fewer physical and psychological symptoms, and a better quality of life.

Table 3 illustrates the results of the bivariate analysis of the disparities between being/not being a victim of bullying with regard to the aforementioned psychosocial variables. This analysis focused solely on the subset of students with CC. All variables investigated exhibited statistically significant differences.

The results demonstrated that adolescents with CC who were victims of bullying were more involved in violent behavior as aggressors (bullying and cyberbullying) and were more victims of cyberbullying compared to adolescents with CC who were not victims of bullying. They also liked school less, engaged more frequently in self-harm behaviors, and demonstrated higher levels of anxiety and more depressive symptoms. Adolescents with CC who were not victims of bullying reported receiving more significant support from family and friends, having better relationships with teachers and peers, and experiencing fewer physical and psychological symptoms, and better quality of life.

Table 4 presents the results of the multiple linear regression model of the variables for the study of the quality of life of adolescents with CC. The model was adjusted to age and gender and explained 57% of the variance in the quality of life of adolescents with CC, *F*(15,680) = 61,40; *p*≤0.001.

**Table 4:** Multiple Linear Regression Model of Variables to Study the Quality of Life of Adolescents With CC.


	UNSTANDARDIZED COEFFICIENT		STANDARDIZED COEFFICIENT	*t*
	
	B	STANDARD ERROR	*β*	

Being a bully	0.93	0.62	0.04	1.50

Being a victim of bullying	–1.07	0.52	–0.06*	–2.07

Being a cyberbully	–2.05	0.89	–0.06*	–2.29

Being a victim of cyberbullying	0.58	0.63	0.03	0.92

Liking school	1.12	0.41	0.08**	2.71

Self-harm	0.17	0.03	0.16***	5.24

Family support	0.13	0.03	0.12***	3.87

Support from friends	0.11	0.09	0.04	1.26

Relationship with teachers	0.17	0.08	0.06*	2.08

Relationship with peers	–0.06	0.47	–0.00	–0.14

Physical and psychological symptoms	0.12	0.03	0.13***	3.93

Anxiety	–0.47	0.09	–0.18***	–5.13

Depressive symptoms	–0.33	0.05	–0.28***	–7.23


The results were adjusted for age and gender. The variables were entered using the “enter” mode. *p ≤ 0.05. **p ≤ 0.01. *** *p* ≤ 0.001. Dependent variable: Perception of quality of life.

As illustrated, being a victim of bullying, being a cyberbully, and demonstrating anxiety and depressive symptoms were negatively associated with quality of life. By contrast, liking school, receiving support from family and friends, having relationships with peers, and physical and psychological symptoms were positively associated with quality of life. Thus, a better quality of life was associated with not being a victim of bullying, not being a cyberbully, having less anxiety and fewer depressive symptoms, liking school, and having more support from both family and friends, better relationships with peers and fewer physical and psychological symptoms.

## Discussion

The findings reveal that adolescents with CC face more significant psychosocial challenges than their peers without CC. They not only experience more involvement in situations of violence, such as being victims of bullying and cyberbullying, and engage in self-harm behaviors more frequently but also report heightened emotional struggles, including increased anxiety levels and more depressive symptoms. Conversely, adolescents without CC indicate stronger family support, fewer physical and psychological symptoms, and a better quality of life.

These results mirror evidence found in the literature: Adolescents with CC present increased risks in terms of mental health, psychosocial functioning, and peer victimization (Butler et al., 2018; Cerqueira, Gaspar, et al., 2022; Cerqueira, Guedes, Gaspar et al., 2022; Gaspar et al., 2019; James et al., 2022; Malkowska-Szkutnik & Mazur, 2019; Mullins et al., 2017; Pinquart, 2017; Runions et al., 2020; Sentenac et al., 2022; van der Sprenkel et al., 2022).

The present study also revealed that in addition to the increased risk associated with the existence of a CC, being a victim of bullying increased the psychosocial vulnerabilities. On the one hand, adolescents with CC who were victims of bullying reported more frequently being victims of cyberbullying and engaging in more self-harm behaviors than peers who were not victims of bullying. On the other hand, adolescents with CC who were victims of bullying also reported more bullying and cyberbullying behaviors (as aggressors in both forms of violence and as victims of cyberbullying) than adolescents with CC who were not victims of bullying.

In short, adolescents with CC who are victims of bullying present an increased risk of becoming involved in other forms of violence (i.e., victims of cyberbullying and self-harm) and of perpetuating these behaviors as aggressors (in situations of bullying and cyberbullying). These findings are also in line with the literature supporting evidence suggesting the involvement of students with CC not only as victims but also as aggressors in bullying situations (Beckman et al., 2020; Haegele & Zhu, 2023; Pinquart, 2017; Rupp et al., 2019).

González-Cabrera et al. (2020) found that being a victim of both bullying and cyberbullying results in more negative consequences for quality of life than being a victim of just one of the two types of violence. According to Chudal et al. (2021), the combination of victimization in both types of violence leads to more internalizing symptoms. Therefore, it is essential to consider the overlap that may exist between victimization of bullying and cyberbullying when carrying out interventions in the school context (Pichel et al., 2021).

A lower risk of victimization in bullying situations has been found to be associated with better relationships in the family and the peer group and a greater connection with school (Biswas et al., 2022; Thornberg et al., 2022), reinforcing the importance of interpersonal relationships. These results are in line with those obtained in the present study, as adolescents with CC who were not victims of bullying reported having more significant support from family and friends and a better relationship with teachers and peers. Therefore, promoting socio-emotional skills among students, educators, and families emerges as a crucial protective factor for adolescent well-being (Larrier et al., 2022).

Regarding the psychosocial factors that contribute to explaining the quality of life of adolescents with CC, the results of this study indicate that a better quality of life is associated with not being a victim of bullying, not being a cyberbully, having less anxiety and fewer depressive symptoms, liking school, having more support from both family and friends, better relationships with colleagues, and fewer physical and psychological symptoms.

Bullying is a recurring phenomenon in schools nowadays, and evidence points to the harmful consequences of bullying on the psychosocial development and well-being of adolescents (Arslan et al., 2021; Chudal et al., 2021; Eyuboglu et al., 2021; González-Cabrera et al., 2020; Jackson et al., 2019; Koyanagi et al., 2019; Li et al., 2022). For example, a study by Skarstein et al. (2020) revealed that social exclusion and bullying harmed the quality of life of adolescents with CC. Thus, participants in the study described feelings of sadness, loneliness, and depression related to the instances of bullying and social exclusion they had experienced. Similarly, Sentenac et al. (2022) noted that students with CC have higher levels of negative school experiences (including peer victimization) and that this is related to lower life satisfaction.

Given the evidence suggesting that there is a relationship between more positive school environments and less student involvement in bullying situations (Dorio et al., 2020; Farina, 2019; Hultin et al., 2021; Marchante et al., 2022; Montero-Carretero et al., 2021; Yang et al., 2020), we recommend that future research explore factors in the school context that can positively influence the well-being and participation of students with CC. The experience of a CC constitutes a risk factor for the well-being of adolescents, and the literature reinforces the even more significant impact that occurs when these health conditions affect students’ school participation (Cerqueira, Gaspar, et al., 2022; Cerqueira, Guedes, Gaspar et al., 2022; Cerqueira, Guedes, Marques-Pinto et al., 2022). Therefore, it is essential to involve students, teaching and non-teaching staff, families, and the community in general in collaborative efforts to promote healthy and positive environments.

This study had some limitations that must be acknowledged: (a) the self-reporting nature of the data may lead to some bias (e.g., memory bias); (b) participants were students attending the public school system; that is, students attending the private school system and students who are not attending an educational institution (for various reasons) are not represented, limiting the generalizability of the findings; (c) the study’s cross-sectional design hampered our ability to infer causality and to ascertain the direction of effects. Thus, any generalizations made should acknowledge this constraint. To overcome this challenge, longitudinal data would be necessary; (d) constraints were imposed as a result of utilizing pre-existing datasets such as the HBSC study. While this approach offers valuable insights, it also restricts the selection of items available to represent certain constructs. For instance, although the anxiety scale’s alpha coefficient may not reach high levels, it is a validated scale within the HBSC protocol specifically designed for the adolescent population. Nevertheless, the HBSC study presents several strengths: it follows a rigorous methodology, includes a large representative sample of Portuguese adolescents, and allows comparisons between the different years of the study (HBSC is developed every four years) and the different countries involved.

## Conclusion

The level of psychosocial risk differs significantly between adolescents with and without CC, making it vital to consider the increased challenges associated with the existence of a chronic health condition. The results of the current study underline the existence of worse results in terms of psychological and social variables in adolescents with CC. Specifically, a CC constitutes a risk factor for the psychosocial well-being and quality of life of adolescents, and experiencing a victimization by peers places these adolescents in an even more vulnerable situation.

Another aspect to take into consideration is the fact that adolescents with CC who are victims of bullying more frequently report being victims of cyberbullying than adolescents with CC who are not victims of bullying. Hence, this study’s findings significantly emphasize the importance of addressing violence in school contexts through tailored strategies and interventions.

Strengthening support networks for students with CC is critical for their quality of life and general welfare. This must involve engaging all agents in the educational context in constructing school environments that promote the psychosocial well-being of all students. Given the increasingly intensive use of digital media, it is important to emphasize that the percentage of adolescents with CC in the current study who reported being a cyberbully and a victim of cyberbullying was higher among those who also reported being victims of bullying. Therefore, it is important not to lose sight of the possible negative cumulative effects of being a victim/aggressor or a victim/victim.

The findings of the present study yields significant insights for health and education professionals and clues for public policies regarding the increased risk that exists in the adolescent population with CC regarding bullying and cyberbullying and about helping schools develop effective tools and strategies to address violence and support students with CC. This group of students is at a higher risk of being involved in such situations and requires a targeted response. It is fundamental to promote the involvement of teaching and non-teaching staff and families in this journey to tackle violence and invest in raising awareness of the increased psychosocial risks that exist in adolescents with CC.

In short, the major findings are the following:

– Adolescents with CC face a higher risk of experiencing violence, both in person and online, underscoring the need for violence-free school environments;
– Adolescents with CC often are faced with heightened emotional challenges;
– Adolescents with CC who are victims of bullying tend to have weak support networks;
– Promoting academic and social participation and empowerment of adolescents with CC is essential to moving toward a more inclusive, balanced, and healthy society.

As recommendations and implications for practice, this study highlights the importance of (a) implementing programs fostering personal and socio-emotional skills to enhance positive relationships and well-being among students, educational teams and families; (b) developing assessment tools focusing on the school environment as a supportive and health-promoting context; (c) encouraging active participation of students, teaching and non-teaching staff, and families in initiatives fostering inclusivity/integration and interpersonal relationships; (d) facilitating extracurricular activities promoting communication, collaboration, and cohesion within the school community; (e) conducting tailored awareness and health promotion activities addressing bullying and cyberbullying, especially for at-risk groups like students with CC; and (f) enhancing communication among education professionals, health professionals, and families to bolster support networks and collaborative efforts for the well-being of adolescents with CC.
